# MCR-3 and MCR-9 confer species-specific increases in colistin MICs

**DOI:** 10.1099/mic.0.001759

**Published:** 2026-08-19

**Keywords:** colistin resistance, *Enterobacteriaceae*, *mcr*

## Abstract

For the clinical treatment of infections with *Enterobacteriaceae*, the development of resistance to last-resort antimicrobials like colistin is of concern, as it limits treatment options. Since 2015, ten families of *mobile colistin resistance* (*mcr*) genes have been discovered; however, our understanding of MCR remains limited because (i) *mcr-1* is the most studied variant, (ii) *mcr* variants are rarely compared and (iii) *mcr* variants are primarily studied in *Escherichia coli*, even though studies have shown that *mcr* variants can confer significantly different phenotypes and that lipid A (i.e. the target of colistin and *mcr-*modification) structurally differs between bacterial species. To fill this gap, we examined how *mcr-3* and *mcr-9*, as two less frequently studied *mcr* variants, impact the colistin resistance of laboratory-adapted and real-world (i.e. clinical or food) isolates of *E. coli*, *Salmonella enterica* and *Klebsiella pneumoniae*. We found that while *mcr-3* consistently conferred colistin resistance to all tested strains, *mcr-9* only conferred resistance to two strains. The fold-change in colistin minimum inhibitory concentrations was significantly impacted by both the bacterial species and the *mcr* variant but not by whether a strain was a laboratory-adapted or real-world isolate. Overall, our results suggest that while laboratory-adapted strains may provide a good estimate of *mcr*-mediated colistin resistance of real-world isolates, findings of *mcr*-mediated phenotypes in one bacterial species should not be extrapolated to another.

## Data summary

The authors confirm all supporting data, code and protocols have been provided within the article or through supplementary data files.

## Introduction

Antimicrobial resistance remains one of the top global public health threats, according to the World Health Organization (WHO) [1]. In the USA, the Centers for Disease Control and Prevention (CDC) have defined carbapenem-resistant *Enterobacteriaceae* (CRE) as an urgent threat [2] whose incidence has been increasing [3]. One of the treatment options for infections with CRE is colistin, a last-resort antimicrobial peptide with strong side effects [4, 5]. Unfortunately, *mobile colistin resistance* (*mcr*) genes, a group of genes that confer resistance to colistin, have been shown to be circulating among *Enterobacteriaceae* globally [6, 7].

As of early 2026, a total of 10 different *mcr* families have been discovered [7, 8]. All *mcr* genes encode phosphoethanolamine (pEtN) transferases, which transfer a pEtN residue to at least one of the two phosphate groups on lipid A [8]. It is thought that pEtN interferes with colistin’s ability to electrostatically interact with the phosphate groups on lipid A, thus conferring colistin resistance [8, 9]. While the enzymatic action of all MCR variants is similar, there are considerable differences in the amino acid sequences [10]. Additionally, the colistin MICs conferred by each MCR variant also appear to differ [11, 12]. We recently showed [12] that MCR-1 and -3 modify the 4-phosphate, while MCR-9 modifies the 1′-phosphate of lipid A, which may explain differences in phenotypes conferred by different MCR variants. Despite evidence that MCR variants confer distinct phenotypes, studies still primarily focus on *mcr-1*. And while lipid A can structurally differ between bacterial species [13, 14], most studies have used the same model organism – *Escherichia coli* – to study the effects of *mcr*-mediated lipid A modifications.

Because the interactions between colistin and lipid A are instrumental for colistin resistance, we hypothesized that the colistin resistance conferred by MCR variants is dependent on (i) the MCR variant and (ii) the bacterium expressing MCR. To test this hypothesis, we examined how *mcr-3* and *mcr-9* impact the colistin resistance of laboratory and real-world isolates of three different *Enterobacteriaceae* species: *E. coli*, *Salmonella enterica* subsp. *enterica* serovar (*S*.) Typhimurium and *Klebsiella pneumoniae*.

## Methods

### Bacterial strains and growth conditions

All bacteria and plasmids used in this study can be found in Table 1 and were grown at 37 °C, 200 r.p.m. in Difco LB Lennox Broth [LB; Becton, Dickinson and Company (BD), Franklin Lakes, NJ, USA]. Strains with the integrated *attTn7* transposons were grown with the supplement of 30 (*E. coli* and *S*. Typhimurium) or 100 (*K. pneumoniae*) μg ml^−1^ of chloramphenicol (CM). *E. coli* MFD strains were grown in 0.5 mM 2,6-diaminopimelic acid (DAP; Thermo Fisher Scientific; Waltham, MA, USA) and pTnS2 plasmid-carrying strains were grown in 100 µg ml^−1^ ampicillin.

**Table 1.** Bacterial strains, plasmids and their sources used in this study

Strain	FSL ID*[Table-fn T1_FN1]	Note	Source
*E. coli* MG1655	S14-0029	Laboratory strain	Peters Laboratory, Cornell University
*E. coli* O157:H7	F6-1038	Human clinical isolate	FSL strain collection
*S*. Typhimurium LT2	S2-6012	Laboratory strain	FSL strain collection
*S*. Typhimurium	R8-2457	Human clinical isolate	FSL strain collection
*K. pneumoniae* 13883	Z3-0206	ATCC standard strain	ATCC (13883)
*K. pneumoniae*	T4-0715	Ready-to-eat food isolate	FSL strain collection
*E. coli* Pir1	NA	For cloning of plasmids	Thermo Fisher
*E. coli* MFD	S14-0121	For transposon transposition	Doerr Laboratory, Cornell University
**Plasmid**			
pTnS2	NA	Plasmid containing Tn7 transposase	[33]
pGP-Tn7-pCYM	NA	Plasmid containing cumate-inducible promoter on Tn7 transposon	[18]
pGP-Tn7-mcr-3	NA	Plasmid containing cumate-controlled *mcr-3* expression transposon	This study
pGP-Tn7-mcr-9	NA	Plasmid containing cumate-controlled *mcr-9* expression transposon	This study

* FSL IDs are our lab internal ‘Food Safety Laboratory’ IDs. More information about these strains can be found on foodmicrobetracker.net.

### Construction of inducible *mcr-3* and *mcr-9* in different bacterial strains

*Mcr-3.1* (from *S.* Typhimurium FSL R9-3269, SRA: SRS1245499 [15]) and *mcr-9.1* (NCBI accession no. NZ_NAAN01000063.1, NCBI protein accession no. WP_001572373.1) with 3x-FLAG tag were cloned from previously created pBAD24 plasmids [12] using the forward primers pGP-mcr-3_f (5′ CGTAGAATTCATGATGCAGCATACTTC 3′), pGP-mcr-9_f (5′ CGTAGAATTCATGTTTTTACTGGTTTACTAT 3′), the reverse primer pGP-FLAG_r (5′ CCTAGGGCCCTTATTTATCATCATCATC 3′) and the Q5 high-fidelity DNA polymerase [New England Biolabs (NEB); Ipswich, MA, USA]. The pGP-Tn7-pCYM plasmid and the PCR products were digested with EcoRI-HF and ApaI (NEB), ligated with DNA ligase (NEB) and transformed into *E. coli* Pir1 strains via heat shock, according to the manufacturer’s recommendations. The correct insertion of genes was confirmed via Sanger sequencing with the pUC18R6KT_seq_f (5′ TTCACTTATCTGGTTGGCC 3′) and pGP_seq_r (5′ CGGTTCGTAAACTGTAATG 3′) primers, performed at the Biotechnology Resource Center (BRC, Cornell University, Ithaca, NY). Correctly assembled plasmids were transformed into competent *E. coli* MFD via heat shock. To allow *Tn7-pCYM* (‘empty’), *Tn7-mcr-3* (‘*mcr-3’*) and *Tn7-mcr-9* (‘*mcr-9*’) to insert into the *attTn7* site, the recipient strains (*E. coli*, *S*. Typhimurium and *K. pneumoniae*, see Table 1) were mated with *E. coli* MFD pTnS2 and *E. coli* MFD pGP-Tn7 (empty, *mcr-3* and *mcr-9*) strains in 3 : 1 : 1 ratios on LB + 0.5 mM DAP plates for 6 h. Afterwards, the conjugation mixture was removed from the plates using a sterile loop, washed in PBS and plated on LB plates supplemented with CM. After 18–24 h of incubation, colonies were picked and screened with attachment site spanning primers AG67_148 (5′ NNNNATAAAACGAAAGGCCCAGTCTTTCGACTGAGCCTTTCGTTTTATTTG 3′) and attTn7_seq_r (5′ GACCAGCCGCGTAACCTG 3′). All positive strains were also screened with the pGP-mcr-3_f, pGP-mcr-9_f and pGP-FLAG_r primers.

### Determination of optimum cumate concentration

To determine the optimum cumate inducer concentration, *E. coli* MG1655 strains with integrated Tn7 transposons (empty, *mcr-3* and *mcr-9*) were tested for (i) colistin susceptibility, (ii) growth and (iii) protein expression (through Western blots) when induced with 0, 10, 50 or 100 µM of 4-Isopropylbenzoic acid (in the following referred to as cumate; Sigma-Aldrich; St. Louis, MO, USA). Susceptibility testing, growth curves and Western blots were performed as described below. Each strain and cumate concentration was tested in biological triplicate.

### Growth curves

Growth patterns of *mcr*-expressing bacterial strains were determined by measuring the optical density at 600 nm (OD_600_) as previously described [16]. Briefly, each strain was grown overnight, backdiluted 1 : 200 into Mueller–Hinton II (MHII, BD) broth containing appropriate antibiotics and 10 µM of cumate (except for optimum cumate concentration determination). Growth was assessed in triplicates and duplicates in the presence and absence of cumate, respectively. Strains were incubated at 37 °C for 24 h with continuous shaking in 96-well, clear flat-bottom polystyrene plates (Corning; Corning, NY, USA) in the Synergy H1 Microplate Reader (BioTek Instruments; Winooski, VT, USA). OD_600_ readings were taken every 10 min. The OD_600_ of every strain was obtained by subtracting the background OD_600_ readings of the MHII media controls.

### Susceptibility testing

Susceptibility testing was performed as previously described [12]. Briefly, each strain was grown for 16–20 h and backdiluted 1 : 200 into MHII containing appropriate levels of CM. After 2 h of incubation, 10 µM of cumate was added and cultures were incubated for another hour. Cultures were then inoculated in MHII broth containing colistin (0.25–16 µg ml^−1^, twofold dilutions) to achieve final concentrations of 5×10^5^ c.f.u. ml^−1^ and incubated statically at 35 °C for 16–20 h. All strains were assessed in biological quadruplicates.

### Western blot analysis

To confirm MCR-3- and MCR-9-FLAG expression, strains were grown for 16–20 h, backdiluted 1 : 200 in MHII containing appropriate levels of CM and incubated for 2 h. Afterwards, 10 µM of cumate was added and strains were incubated for 6 h. Cultures were collected and processed for Western blot detection using anti-FLAG-rabbit (Sigma-Aldrich, 1 : 500) and anti-rabbit-goat-horseradish (Thermo Fisher Scientific, 1 : 5,000) as previously described [17].

### Statistical analysis

Statistical analyses were performed using R Statistical Software (Version 4.1.2; R Foundation for Statistical Computing, Vienna, Austria). Means of log_10_-transformed MCR protein levels were compared by a one-way ANOVA with post-hoc Tukey test. The effects of ‘Strain’ (i.e. bacterial species), ‘*mcr* variant’ (i.e. *mcr-3* or *mcr-9*) and ‘type’ (i.e. laboratory-adapted or real-world isolate) on log_10_-transformed fold-changes in MIC were assessed with an ANOVA with an interaction between ‘Strain’ and ‘*mcr*-variant’.

## Results and discussion

### Determining an optimum cumate concentration for MCR expression in *Enterobacteriaceae*

**Fig. 1.**
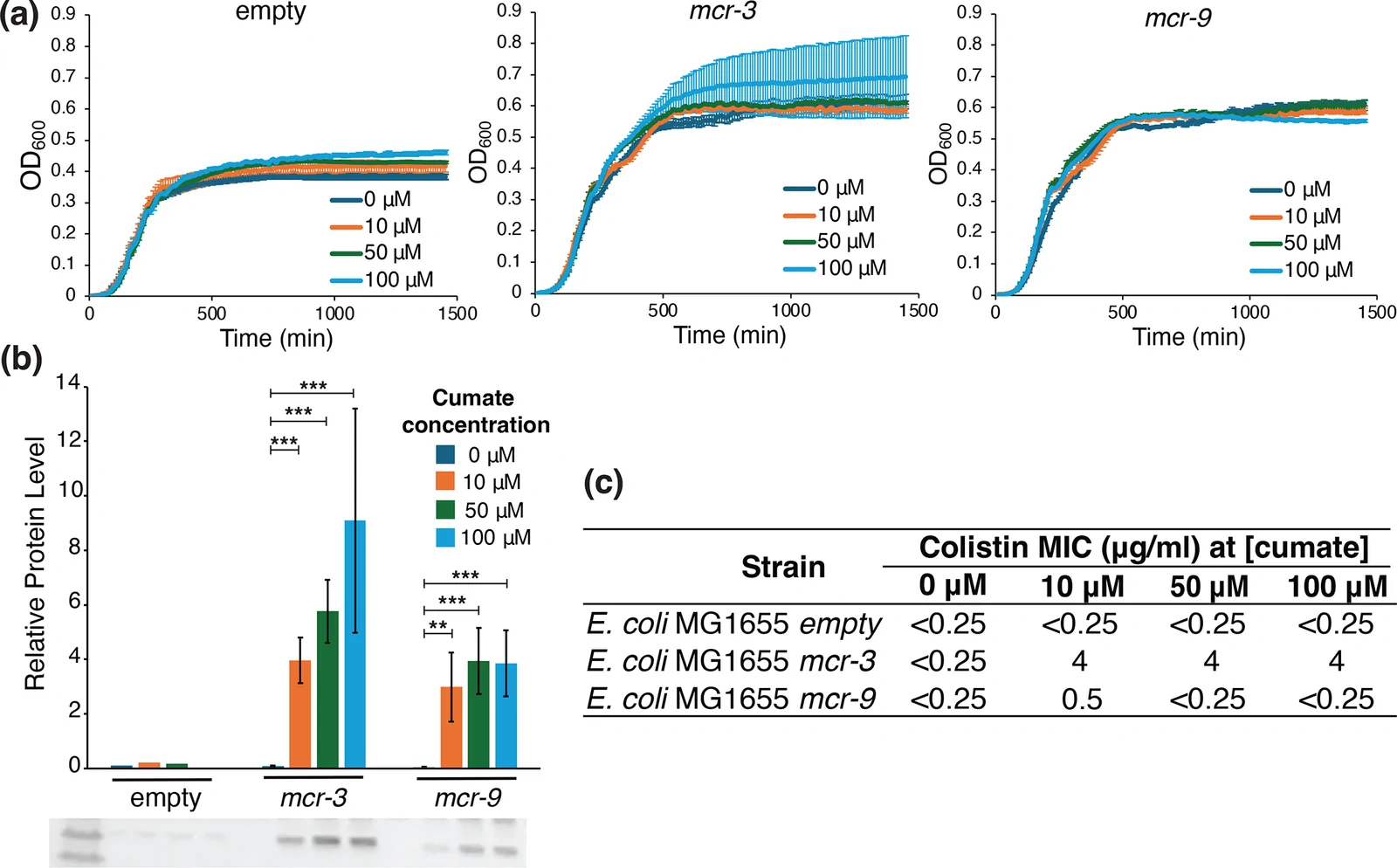
Bacterial growth, MCR expression and colistin MICs of *E. coli* MG1655 carrying the *attTn7* transposons after induction with four different cumate concentrations. (**a**) Absorbance (OD_600_) of *E. coli* MG1655 strains carrying the *attTn7* transposon (empty, *mcr-3* or *mcr-9*) was measured over 24 h in the presence of 0, 10, 50 or 100 µM of cumate. Data represent means, *n*=3±sem. (**b**) Relative protein expression of FLAG-tagged proteins after 2 h of initial growth followed by 6 h of induction with cumate. Data represent means, *n*=3±sem. Significance values are based on comparing log_10_-transformed MCR-3 or MCR-9 levels based on cumate concentrations using a one-way ANOVA with post-hoc Tukey test. Full pictures of SDS-PAGE gels and Western blots that were used to assess relative protein levels can be found in Fig. S1. (**c**) Colistin MICs were obtained after growth in MHII for 2 h, followed by induction with 0, 10, 50 or 100 µM cumate for 1 h. MIC assays were performed in biological triplicate; all replicates had the same MIC. **P<0.01; ***P<0.001.

To determine whether MCR variants confer distinct phenotypes in different *Enterobacteriaceae*, we cloned single-copy FLAG-tagged *mcr-3* and *mcr-9* into laboratory strains and clinical or food isolates of *E. coli*, *S*. Typhimurium and *K. pneumoniae* under the control of a cumate-inducible promoter [18]. To evaluate which concentration of cumate is optimal to induce MCR expression, we assessed the growth, protein expression and colistin MICs of *E. coli* MG1655 strains, as the colistin MICs conferred by MCR-3 and -9 are well known in *E. coli* [11, 12]. We chose to assess 0, 10, 50 and 100 µM of cumate (Fig. 1), as similar concentrations were previously shown to successfully express GFP in *E. coli* [18].

When observing the growth of *E. coli* MG1655 *mcr-3* and *mcr-9* strains in the presence or absence of cumate, there were no considerable differences in the OD_600_ of either strain when induced with 10, 50 or 100 µM of cumate in comparison to the uninduced control (Fig. 1a). Similarly, the control strain carrying an empty Tn7 transposon showed no differences in OD_600_ between strains grown in the absence or in the presence of 10, 50 or 100 µM of cumate (Fig. 1a); however, this strain reached a lower maximum OD_600_ (0.4) than the *mcr-3* and *mcr-9* strains (>0.5). While it is unclear why the empty Tn7 strain had a lower maximum OD_600_ than the *mcr-3* and *-9* carrying strains, our results suggest that (i) cumate is not toxic to bacterial cells that carry an empty expression system and that (ii) there is no evidence of toxicity stemming from MCR-3 or MCR-9 expression at the cumate concentrations tested here. These results suggest that the single-copy, cumate-inducible system is appropriate for the expression of MCR variants, which are known to be toxic [8, 12], especially considering that a previous study [12] showed that cell numbers of a *mcr-9-*expressing strain were significantly reduced when grown in the presence of higher inducer concentrations when using a l-aminoarabinose inducible plasmid-based system. Decreases in cell numbers and other fitness indicators in bacteria expressing *mcr* variants are frequently reported by studies using multi-copy plasmid-inducible systems but not in studies utilizing low- or single-copy plasmids [8], suggesting that it is possible that the single-copy nature of the Tn7 system used here prevents potential plasmid-associated fitness costs of *mcr* expression, rather than the nature of the inducible promoter.

We also assessed the relative MCR protein level at each tested cumate concentration (i.e. 0, 10, 50 and 100 µM). As expected, we detected scarcely any protein expression for the control strain with the empty Tn7 transposon. We did not detect any protein expression in the absence of the inducer (Fig. 1b) and the MCR-3 and -9 protein levels were significantly higher (*P*<0.01, ANOVA with post-hoc Tukey test) when grown in the presence of the inducer (i.e. 10, 50 or 100 µM) than when grown in the absence of inducer. The MCR-3 or -9 protein levels did not significantly differ (*P*>0.05, ANOVA with post-hoc Tukey test) when induced with different concentrations (i.e. 10, 50 or 100 µM) of cumate (Fig. 1b). Interestingly, we did not detect any protein expression when strains were grown in the absence of cumate, suggesting tight control of the cumate-inducible promoter. This is in contrast to other expression systems, e.g. systems using arabinose- or lactose-inducible promoters, which are leaky and oftentimes express protein even in the absence of the inducer [16, 17, 19]. This difference may be because expression from the cumate promoter is induced when cumate is added, while expression from the arabinose promoter is de-repressed when l-aminoarabinose is added [18].

Lastly, we assessed the colistin MICs of *E. coli* MG1655 strains expressing MCR-3 and -9, in comparison to the control carrying an empty Tn7 transposon (Fig. 1c). When uninduced, all three strains have the same MIC (i.e. <0.25 µg ml^−1^) which is similar to colistin MICs reported for *E. coli* MG1655 and other *E. coli* K-12 strains, i.e. <0.5 µg ml^−1^ [17]. This further exemplifies that the cumate-inducible promoter allows for tight regulation of protein expression. The colistin MICs of *E. coli* MG1655 *mcr-3* were the same (i.e. 4 µg ml^−1^) when induced with 10, 50 and 100 µM of the cumate (Fig. 1c). In contrast, the colistin MIC for *E. coli* MG1655 *mcr-9* was 0.5 µg ml^−1^ when induced with 10 µM of colistin but <0.25 µg ml^−1^ when induced with 50 or 100 µM of cumate, most likely due to variation (Fig. 1c). The colistin MICs for *E. coli* MG1655 *mcr-3* and *mcr-*9 strains are in line with previously reported colistin MICs for *E. coli* expressing *mcr-3*, i.e. 2 µg ml^−1^ [12, 20] and *mcr-9*, i.e. 0.25 µg ml^−1^ [12, 21, 22], respectively.

Based on our results, we chose to use 10 µM of cumate to induce the expression of MCR-3 and -9 in other *Enterobacteriaceae*. We recognize that expression levels may differ between bacterial species; however, these differences will likely be negligible as our results suggest that higher levels of MCR protein may not necessarily increase colistin MICs.

### Colistin MICs conferred by MCR-3 and MCR-9 differ depending on the bacterial species expressing them

To assess whether MCR variants confer distinct phenotypes in different bacterial species, we cloned the previously assembled cumate-inducible FLAG-tagged *mcr-3* and *mcr-9* constructs within the *Tn7* transposon (i.e. the same constructs that were cloned into *E. coli* MG1655) into the *attTn7* site of five additional strains. Because laboratory strains have evolved to adapt to laboratory conditions, suggesting that it may not always be appropriate to extrapolate findings made with laboratory strains to wildtype and/or clinical strains [23], we cloned *mcr-3* and *-9* into one laboratory or type-strain and one clinical or food isolate (in the following referred to as ‘real-world isolates’) of *E. coli*, *K. pneumoniae* and *S*. Typhimurium (see Table 1). Concerns for the applicability of results are especially high when working with *E. coli* MG1655 (as well as other *E. coli* K-12 strains) as these strains do not express an O-antigen [24], which may be important for interactions between colistin and *mcr*-modified lipid A. We want to make it clear that even though we chose a clinical *E. coli* O157:H7 strain for this work, antibiotics are not the recommended treatment for infections with *E. coli* O157:H7 due to the increased risk of developing haemolytic uraemic syndrome [25].

**Fig. 2.**
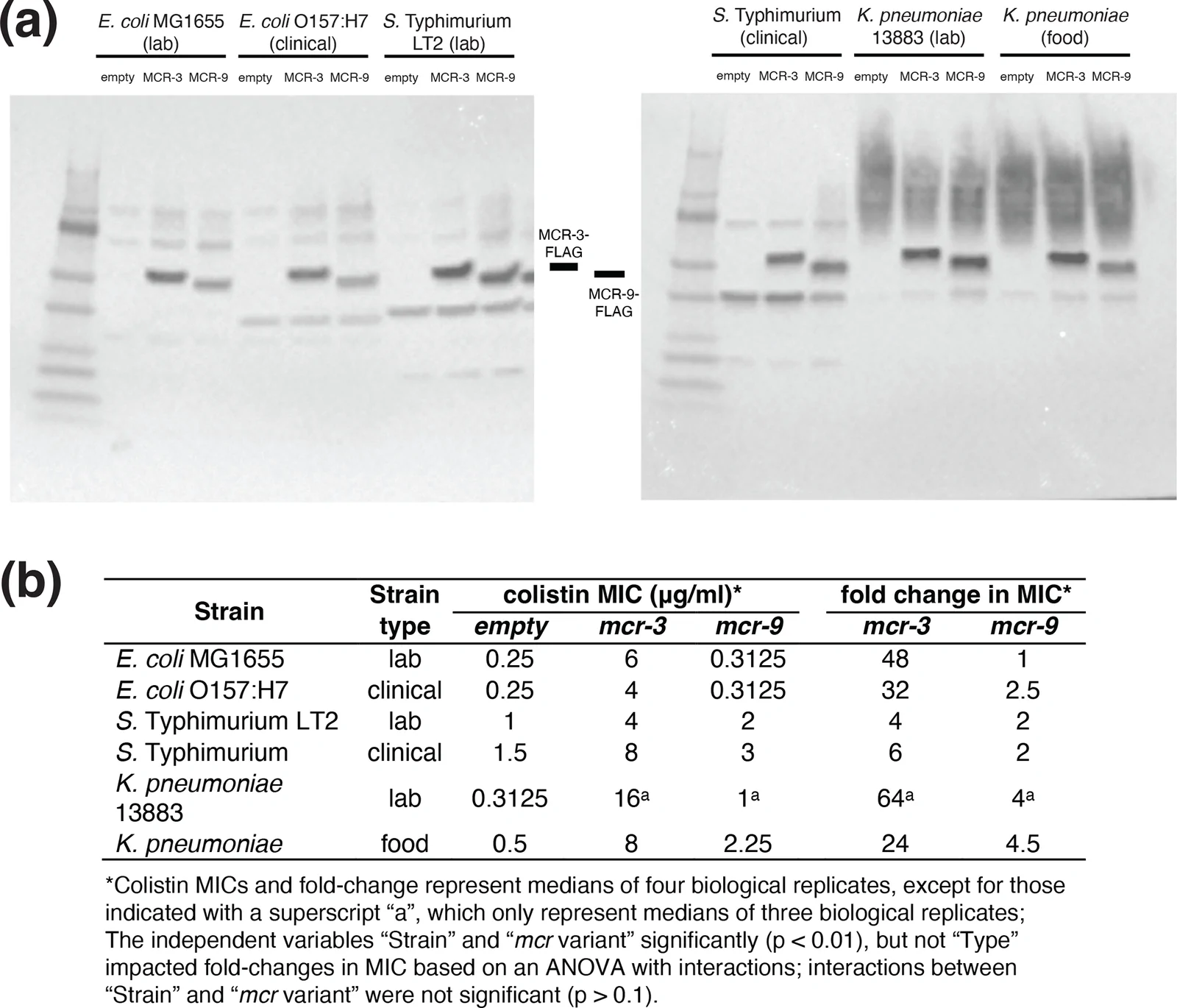
Western blots and MICs of *Enterobacteriaceae* expressing MCR-3 and MCR-9. (**a**) Image of Western blots, probing for MCR-FLAG expression using anti-FLAG and anti-rabbit antibodies of *Enterobacteriaceae* carrying *attTn7::empty, mcr-3* or *mcr-9* after growth for 2 h and induction with 10 µM of cumate for 6 h. The estimated sizes of the MCR-FLAG proteins are shown in between the two Western blot images. (**b**) Colistin MICs of strains induced with 10 µM of cumate are shown as medians of four biological replicates, except those indicated with a superscript a. Fold changes in MICs are based on the MICs of each strain carrying *attTn7::empty*, in comparison to those carrying *attTn7::mcr-3* or *::mcr-9* and represent the median.

**Fig. 3.**
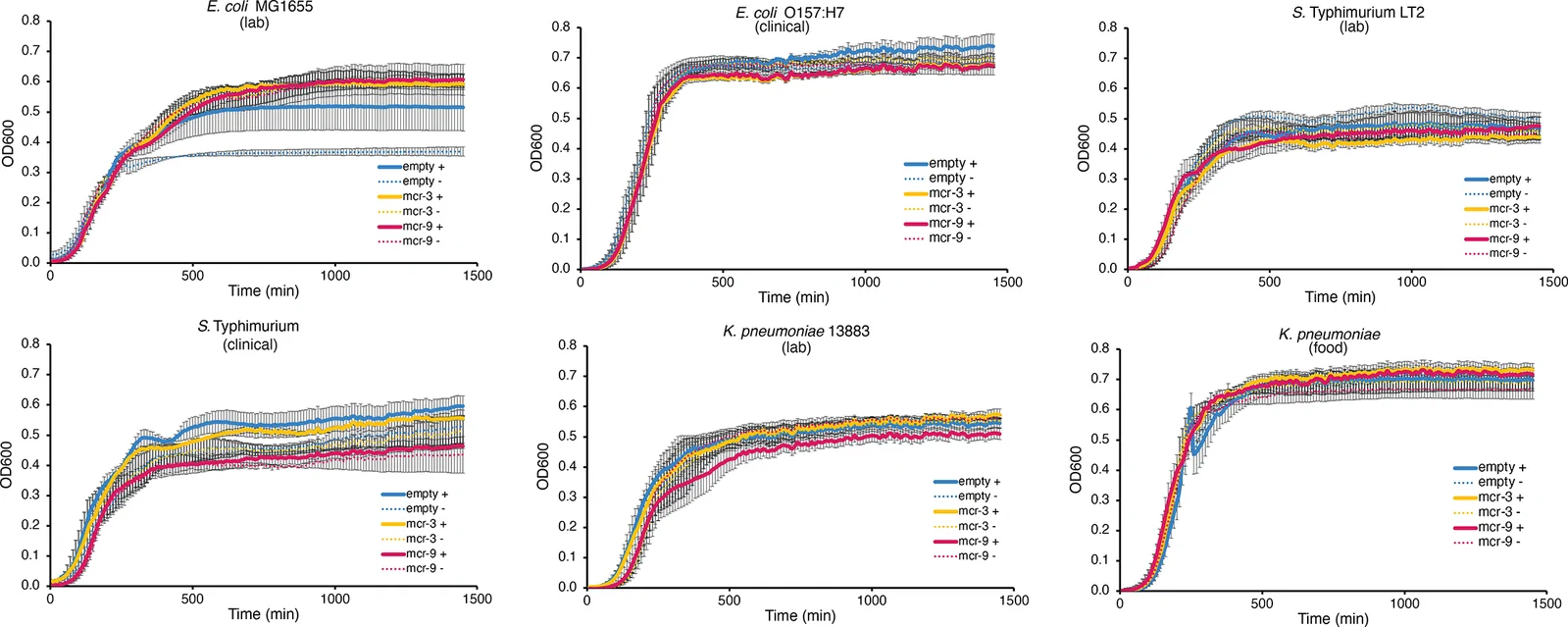
Growth curves of *Enterobacteriaceae* expressing MCR-3 and MCR-9. Absorbance (OD_600_) of strains carrying the attTn7 transposon (empty, mcr-3 or mcr-9) was measured across 24 h in the presence of 0 (indicated by ‘-’ in figure legend) or 100 μM (indicated by ‘+’ in the figure legend) of cumate in biological triplicates and duplicates, respectively.

All six strains (Table 1) were first assessed for their ability to express functional MCR-3 and MCR-9 proteins when induced with 10 µM of cumate (Fig. 2a). Except for the control strains carrying the empty Tn7 transposon, all six bacterial strains (i.e. both laboratory and real-world isolates) expressed functional MCR-3 and -9 (Fig. 2a), showing that the Tn7-located cumate-inducible expression system allows for MCR expression in the three different *Enterobacteriaceae* species tested here. Additionally, when assessing bacterial growth through OD_600_ growth curves, we did not detect considerable differences in OD_600_ values between (i) induced and uninduced and (ii) *mcr-3* or *mcr-9* expressing strains in comparison to the control carrying the empty Tn7 transposon for each strain (Fig. 3). Again, the uninduced *E. coli* MG1655 empty Tn7 strain reached a lower OD_600_ than the *mcr-3* and *-9* carrying strains. For the other strains, we detected only minor differences in OD_600_ values for *mcr-3* and *mcr-9* expressing clinical *S*. Typhimurium isolate and the *mcr-9* expressing laboratory *K. pneumoniae* isolate in comparison to the respective empty control strains. While minor decreases in OD_600_ in *mcr*-expressing strains are to be expected for this family of antimicrobial resistance(AMR) genes [8, 12], these results also suggest that the expression system used here may allow for better comparison of phenotypes associated with AMR genes across different bacterial species in contrast to (i) plasmid-based systems which may have different copy numbers in different bacterial species and (ii) induction systems utilizing sugars (e.g. lactose or arabinose) that may be broken down by bacteria resulting in reduced inducer concentrations over time [18].

The colistin MICs of all control strains carrying the empty Tn7 transposon ranged from 0.25 µg ml^−1^ for *E. coli* MG1655 to 1.5 µg ml^−1^ for the clinical *S*. Typhimurium strain (Fig. 2b), which are all considered below the colistin breakpoint defined by EUCAST (i.e. ≤2 µg ml^−1^) [26, 27]. While the median colistin MIC of *E. coli* MG1655 carrying the empty transposon was the same as previously determined (Fig. 1c), *E. coli* MG1655 expressing *mcr-3* and *mcr-9* had slightly different median colistin MICs. This is due to standard twofold differences in individual replicates which can result in variations in median colistin MIC. While it is interesting that the *S*. Typhimurium strains had higher colistin MICs than the *E. coli* and *K. pneumoniae* strains, a study [28] showed that colistin-susceptible *Salmonella* isolates oftentimes have higher colistin MICs than *E. coli* isolates and that *S*. Typhimurium isolates seem to have higher colistin MICs than other *Salmonella* serovars, even in the absence of resistance determinants like *mcr* variants [29]. These differences may be mediated by differences in the *Salmonella* lipid A structure and modifications [14, 30].

All tested bacterial strains that expressed MCR-3 had colistin MICs that were higher than the EUCAST breakpoint [26, 27], suggesting that at least for the strains tested here, *mcr-3* consistently confers colistin resistance, regardless of the initial colistin MIC of a given parent strain that did not carry a *mcr* gene (Fig. 2b). In contrast, only two strains expressing MCR-9 had colistin MICs above the EUCAST breakpoint [26, 27], suggesting that MCR-9 only conferred resistance in the clinical and food isolates of *S*. Typhimurium and *K. pneumoniae* with MICs of 3 and 2.25 µg ml^−1^, respectively (Fig. 2b). Given that the tested clinical *S*. Typhimurium strain carrying the empty Tn7 had a colistin MIC close to the EUCAST breakpoint of 2 µg ml^−1^ (i.e. 1.5 µg ml^−1^), it is possible that the colistin MIC of the *mcr-9* expressing isolate may not accurately reflect *mcr-9*’s ability to confer colistin resistance but is an artefact mediated by a shift from the higher baseline MIC of the clinical *S*. Typhimurium isolate. Furthermore, it is important to note that while *mcr-9* conferred colistin resistance in the food isolate of *K. pneumoniae*, colistin resistance in *K. pneumoniae* clinical isolates is primarily driven by (i) chromosomal mutations in two component systems (e.g. *pmrAB, phoPQ*, *crrAB*) and regulatory genes (e.g. *mgrB*) or (ii) other *mcr* variants like *mcr-1, -3* and *-8*, which all impact lipid A’s modification patterns [31]; thus, the ability of *mcr-9* to confer colistin resistance in *K. pneumoniae* isolates may not be as clinically relevant as other, more common colistin resistance mechanisms.

Because all parent strains without *mcr* genes had varying initial colistin MICs, we examined the fold-changes in colistin MICs, as it allows for more accurate determination of the impact *mcr-3* and *-9* have on the colistin MICs of the tested bacteria. While MCR-3 expression increased the colistin MICs of all six bacterial strains 4–64-fold, MCR-9 carriage increased the colistin MICs of all six bacterial strains 1–4.5-fold (Fig. 2b). The fold-changes in colistin MICs were significantly (*P*<0.01) impacted by the bacterial species and by the *mcr* variant, however, not by the ‘type’ of strain (i.e. whether the strain was a laboratory or real-world isolate) or the interaction between the bacterial species and the *mcr* variant (*P*>0.05). While it makes biological sense for the interaction between the bacterial species and the expressed *mcr* variant to impact colistin MICs, we likely did not include a large enough number of representatives from each bacterial species to see a measurable interaction.

While utilizing inducible systems to determine the phenotypes conferred by antimicrobial resistance genes has advantages like better comparability across different bacterial strains, it also has drawbacks: expression levels or the regulation of expression may not reflect what occurs in real-world isolates. For example, while *mcr-9* expression conferred colistin resistance in the *S*. Typhimurium isolates, *mcr-9* is frequently found in colistin-susceptible bacterial isolates, including *Salmonella* isolates [21, 32]. Based on previous studies, the *mcr-9* promoter region includes an inverted repeat region which may explain low levels of expression in real-world isolates [22]. While our expression system shows that *mcr-9* can confer colistin resistance to certain strains under optimum expression conditions, these levels of expression may not be reached in real-world isolates [32]. However, circulating *mcr-9* may pose a public health concern if expression levels in *mcr-9*-carrying clinical isolates change.

Overall, our MIC results are in line with prior reports that (i) *mcr-3* seems to consistently confer colistin resistance [8, 11, 12] and that (ii) *mcr-9*, especially in *E. coli* and *S. enterica*, does not always confer resistance to colistin [12, 21]. Our results indicate that fold-change in colistin MIC was not significantly different between laboratory or real-world isolates; colistin MICs were impacted by both the *mcr* variant and the bacterial species that express *mcr*. Our findings are important because they suggest that one should not extrapolate colistin MICs conferred by the same *mcr* variant to different bacterial species. This makes sense as colistin interacts with lipid A which can significantly vary between bacterial species [13]. More importantly, our results suggest that fold-changes in colistin MICs obtained when expressing *mcr* variants in laboratory-adapted strains may be an appropriate estimate of colistin MICs of real-world isolates. The O-antigen potentially may not be instrumental for the interaction between colistin and the bacterial outer membrane in laboratory conditions. However, it is important to recognize that there likely are differences between laboratory and real-world isolates when determining how MCR variants may impact other phenotypes (e.g. virulence, inflammatory response).

## Conclusion

Here, we utilized a Tn7-based, single-copy, cumate-inducible expression system to determine how MCR-3 and MCR-9 impact cell growth and colistin MICs of six laboratory-adapted or real-world isolates representing three different bacterial species. We showed that the cumate-inducible, single-copy system tightly regulates expression of MCR-3 and MCR-9 for the three tested *Enterobacteriaceae* species. Our results also indicate that (i) colistin MICs conferred by the same MCR variant differ between bacterial species, that (ii) MCR-3 consistently conferred resistance to colistin and that (iii) fold changes in colistin MICs obtained for laboratory-adapted isolates may be a good indicator for the ability of a given MCR variant to confer resistance or increased colistin MICs to clinical or food isolates. Our results also suggest that findings of phenotypes conferred by *mcr* variants in one bacterial species should not be extrapolated to other bacterial species. Considering that it is becoming more popular to assign resistance phenotypes based on the presence or absence of AMR genes or mutations, our results also suggest caution when using this practice for all *mcr* variants. While our data suggest that this practice works for *mcr-3* (i.e. the presence of *mcr-*3 is a good indicator that an isolate is resistant to colistin), our data also indicate that for *mcr-9* and potentially other *mcr* variants, resistance phenotypes depend on the bacterial species the *mcr* variant is expressed in. This suggests that the combination of bacterial species and *mcr* variant may have to be taken into consideration when assigning resistance phenotypes bioinformatically.

## Supplementary material

10.1099/mic.0.001759 Fig. S1.

## Abbreviations

mcr: mobile colistin resistance
AMR: antimicrobial resistance
2,6-diaminopimelic acid: DAP
MIC: minimum inhibitory concentration
pEtN: phosphoethanolamine
CRE: carbapenem-resistant Enterobacteriaceae
CM: chloramphenicol
LB: Lennox Broth
MHII: Mueller–Hinton II
