# Supplementary material for: MCR-3 and MCR-9 confer species-specific increases in colistin MICs
Source: Microbiology (Reading). 2026 Aug 19;172(8):001759. doi: 10.1099/mic.0.001759 (PMC13489200; doi:10.1099/mic.0.001759)
Supplement: Supplementary file 1 [file mic-172-01759-s001.pdf]

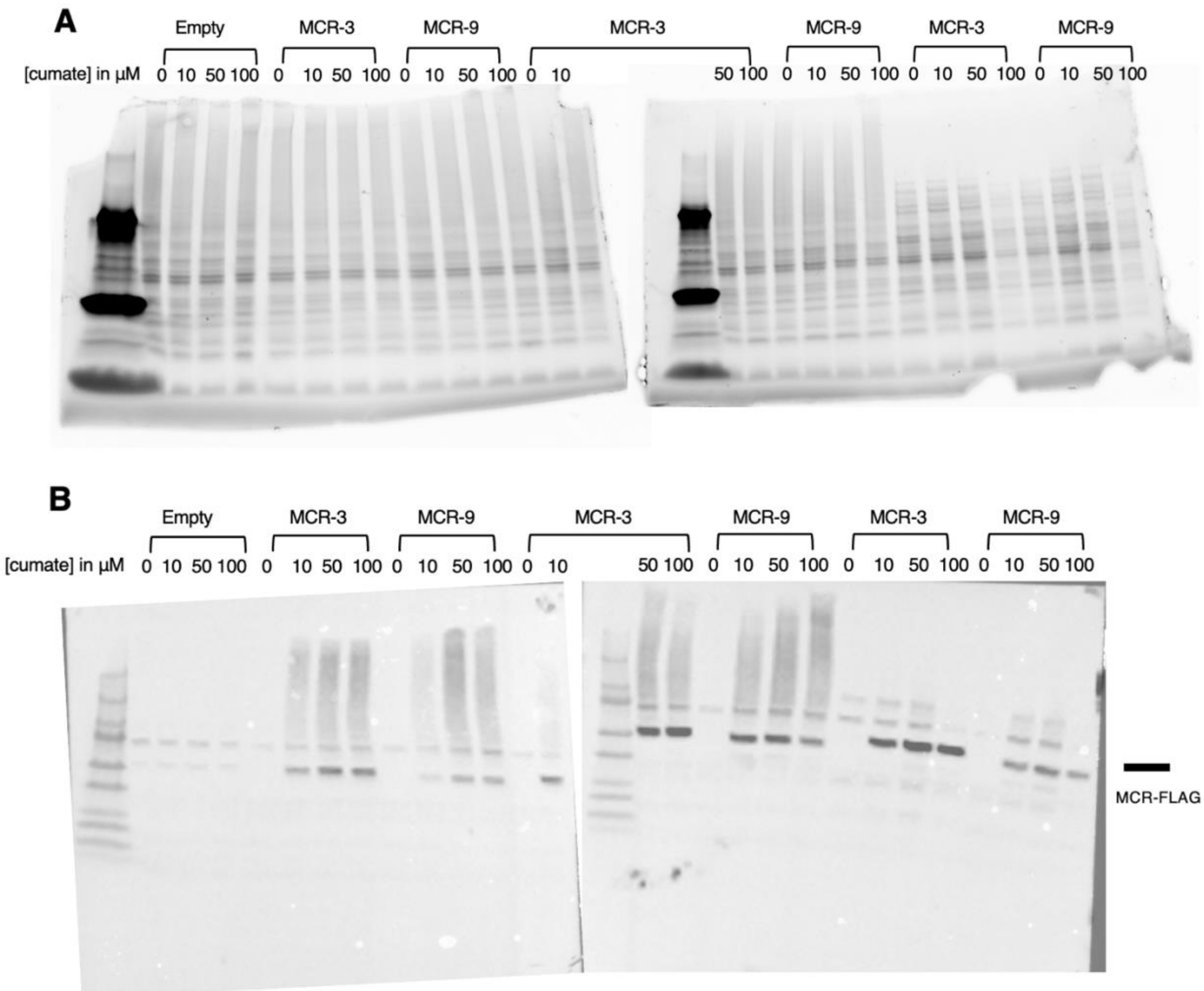

Sup. Figure 1. MCR-3 and MCR-9 protein expression in *E. coli* MG1655. (**A**) SDS page gels and (**B**) Western blots of *E. coli* MG1655 strains used to assess relative MCR-3 and MCR-9 protein expression semi-quantitatively. *E. coli* MG1655 *attTn7::mcr-3* and *::mcr-9* were assessed in biological triplicates, *E. coli* MG1655 *attTn7::empty* was only assessed in one replicate as no protein expression is expected.
